# A comparison between 2 dilute citrate solutions (15mmol/l vs 18 mmol/l) in continuous renal replacement therapy: the base excess and renal replacement solution (BEARRS) study

**DOI:** 10.1186/2197-425X-3-S1-A842

**Published:** 2015-10-01

**Authors:** CM Anstey, AC Richardson, VK Campbell

**Affiliations:** Sunshine Coast Hospital and Health Service, Intensive Care Unit, Nambour, Australia

## Introduction

Regional citrate anticoagulation (RCA) is becoming the preferred method of extracorporeal anticoagulation in patients undergoing continuous renal replacement therapy (CRRT), however the ideal composition remains unclear. We have found protocolised hemofiltration with dilute solutions to be simple and safe, but commercially available solution options are limited. Recently the 14 mmol/L solution (Baxter Haemofiltration Fluid [C14]) was replaced in Australia with 18 mmol/L solution [C18], on the premise that the former provided inadequate anticoagulation. With the use of the C18 solution, a higher incidence of metabolic alkalosis was observed, prompting the design of a biochemically modified solution which would provide optimal anticoagulation, but less biochemical disturbance.

## Objectives

This study primarily aimed to assess the changes in biochemistry occurring in patients undergoing CRRT using two trisodium citrate solutions, the C18 (commercially available in Australia) and a custom manufactured solution containing 15mmol/L (Baxter NamSol [C15]), delivered as a predilution fluid for hemofiltration.

Secondary aims included duration of CRRT, filter life, length of ventilation, length of stay and mortality. The occurrence of significant adverse events was also noted.

## Methods

This is a single-blinded prospective randomised control trial, in a major regional adult intensive care unit. Patients were consented and allocated to one of 2 RCA fluids (C18 or C15) for CRRT. Progress was monitored using a standard daily panel of acid-base and biochemical tests. Analyses were performed days 1, 3 and 5.

## Results

**Figure 1:**
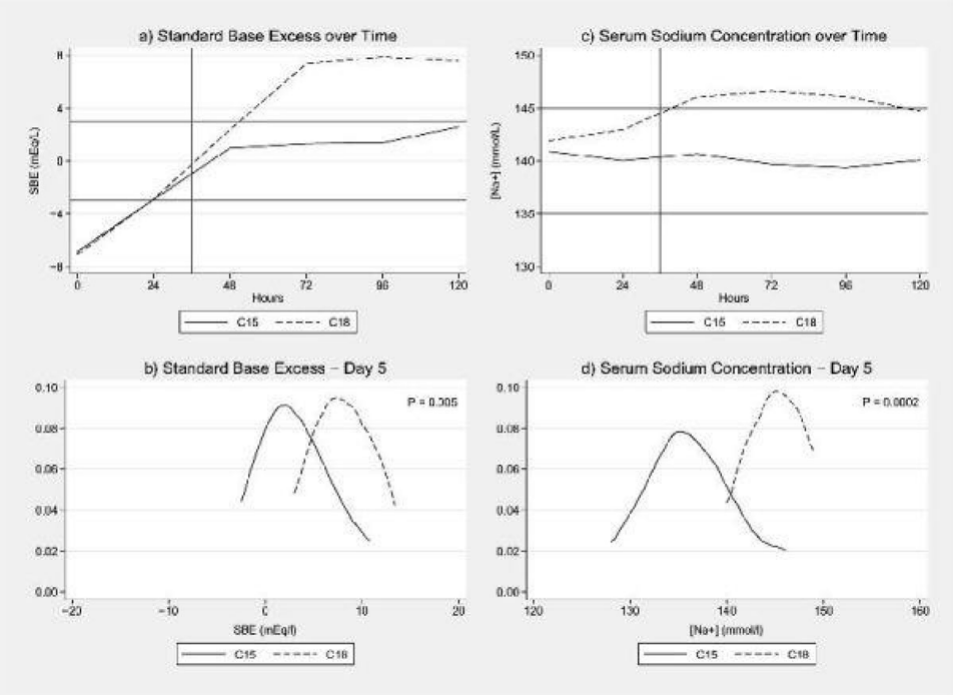
**Data trends of SBE and [Na+].**

In total 48 patients, 23 C18 and 25 C15, were recruited. They were well matched for demography, severity of illness and initial biochemical and acid-base derangement. In both groups, acidosis resolved within 36 hours of institution of CRRT. On day 5 there were significant differences only in [Na^+^] (144.7 mmol/L (SD 4.4) vs 140.1 mmol/L (SD 3.3), p = 0.0002) and Standard Base Excess (SBE) (7.6 (SD 3.5) vs 2.6 (SD 3.2), p = 0.005), both being higher in the C18 group (figure 1).

By Day 5, P_a_CO_2_ had also risen in the C18 group (P = 0.03). Four patients receiving C18 left the study by day 5 due to concerns regarding very high SBEs (> 12 meq/L).

Albeit underpowered, there were no significant differences in secondary end-points, and no significant adverse events were noted in either group.

## Conclusions

For CRRT, the custom C15 solution provided equivalent acidosis recovery and filter-life to the C18 solution, but without the significant hypernatraemia and metabolic alkalosis. Neither solution was associated with any clinically evident adverse events. The C15 solution is a step closer to an ideal RCA replacement fluid, and should be considered for wider availability.

